# Glycated Hemoglobin in the Diagnosis of Diabetes Mellitus in a Semi-Urban Brazilian Population

**DOI:** 10.3390/ijerph16193598

**Published:** 2019-09-26

**Authors:** Nayla Cristina do Vale Moreira, Renan M. Montenegro, Haakon E. Meyer, Bishwajit Bhowmik, Ibrahimu Mdala, Tasnima Siddiquee, Virgínia Oliveira Fernandes, Akhtar Hussain

**Affiliations:** 1 Institute of Health and Society, Department of Community Medicine and Global Health, University of Oslo (UiO), Oslo 0318, Norway; h.e.meyer@medisin.uio.no (H.E.M.); doctorbiplob@yahoo.com (B.B.); 2 Faculty of Medicine, Federal University of Ceará (FAMED-UFC), Fortaleza-Ceará 60020-181, Brazilvirginiafernande@hotmail.com (V.O.F.); hussain.akhtar@nord.no (A.H.); 3 Centre for Global Health Research, Diabetic Association of Bangladesh, Dhaka 1000, Bangladesh; tasnimasiddiquee08@yahoo.com; 4 Institute of Health and Society, Department of General Practice, University of Oslo (UiO), Oslo 0318, Norway; ibrahimu.mdala@medisin.uio.no; 5 Faculty of Health Sciences, Nord University, Bodø 8049, Norway

**Keywords:** diabetes mellitus, glycated hemoglobin, diagnosis, insulin resistance, Brazil

## Abstract

The study evaluated glycated hemoglobin (HbA1c) as a diagnostic tool for diabetes and pre-diabetes in the Brazilian population. Further, the homeostasis model assessment of insulin resistance (HOMA-IR) was also examined against HbA1c values to identify the most suitable cut-off points for HOMA-IR to predict the risk of diabetes. A cross-sectional study was conducted among 714 randomly selected subjects. HbA1c, fasting, and 2 h plasma glucose values were measured. Insulin resistance estimates were calculated with HOMA-IR. The receiver operating characteristic curve assessed HbA1c performance. The adjusted prevalence rate of diabetes mellitus was 14.7%, and pre-diabetes 14.2%. The optimal HbA1c cut-off value was ≥6.8% for the diagnosis of diabetes, and ≥6.0% for pre-diabetes. The area under the curve using HbA1c was 0.85 (95% CI: 0.80–0.90) for detecting diabetes and 0.61 (95% CI: 0.55–0.67) for pre-diabetes. The optimal HOMA-IR cut-off value was 2.06 for HbA1c at 6.8%. The HbA1c cut-off value of ≥6.8% may be suitable for diagnosing diabetes in the Brazilian population. Our results do not support the use of HbA1c to diagnose pre-diabetes. A HOMA-IR cut-off point of 2.06 was a sensitive marker to assess the risk of diabetes.

## 1. Introduction

Diabetes mellitus (DM) and pre-diabetes (impaired glucose tolerance and impaired fasting glucose) are huge-scale pandemics. Estimates from 2015 showed that 415 million adults had diabetes worldwide, and this number is projected to increase to 642 million people by 2040. According to the International Diabetes Federation (IDF), Brazil has an estimated prevalence of DM among adults of approximately 10.2%. Brazil is the country in South America with the highest number of people with the disease [1]. 

Type 2 DM, responsible for approximately 90–95% of those with diabetes, is often asymptomatic in its early stages and can remain undiagnosed for years [2]. Around 193 million people are unaware that they have the condition [1]. Uncontrolled DM is associated with dysfunction and failure of several organs, which may result in blindness, limb amputations, peripheral neuropathy, and kidney failure [3,4,5]. Among those with diabetes, cardiovascular disease (CVD) accounts for about half of the total mortality, and the risk of a first myocardial infarction (MI) is similar to that for reinfarction among nondiabetic patients that suffered a previous MI [6,7]. Insulin resistance (IR) has been addressed as the main pathophysiological link between DM and cardiovascular diseases (CVDs), even in the absence of glucose intolerance for a long time [8]. Pre-diabetes greatly increases the risk of developing type 2 DM and is also associated with the occurrence of CVDs. Thus, the early diagnosis of pre-diabetes and diabetes could reduce long-term complications, healthcare costs, and premature death [9,10,11].

The recommended tests and cut-off points for the diagnosis of DM have been changing over the years. Yet no consensus has been reached on the most effective screening test for its detection. Blood glucose measurements have been applied to define diabetes, either by Fasting Plasma Glucose (FPG) levels or the 2 h values in the 75 g Oral Glucose Tolerance Test (OGTT) [12]. Both tests require patients to fast overnight for at least 8–12 h, and their accuracy may be compromised by patient nonadherence to the fasting period, laboratory error or use of certain medications [13,14]. Although measuring FPG is inexpensive and relatively risk-free, the results may vary substantially within individuals over the long term [13], and must be confirmed with a second test on a subsequent day, in the absence of unequivocal hyperglycemia [12]. On the other hand, OGTT is expensive, time-consuming, labor-intensive, and has shown a low overall test-retest reproducibility [13,15].

Screening or diagnosing DM by using glycated hemoglobin (HbA1c) levels has been extensively discussed [15,16]. In 2008, an International Expert Committee recommended the use of the HbA1c test for diagnosing diabetes, with a threshold of ≥6.5% (48 mmol/mol) [17]. The HbA1c test is more convenient (fasting or timed samples are not required), stable and less variable biologically than FPG or OGTT. In addition, the HbA1c is a better indicator of chronic glycemic levels, has a better index of the risk for long-term complications and is less affected by acute perturbations in glucose levels during periods of stress and illness. Nevertheless, some limitations still persist for using the HbA1c test to diagnose diabetes [12,17]. Haemoglobinopathies or renal failure, as well as laboratory error or the use of certain medications, could influence the accuracy of HbA1c analysis [13]. Moreover, conditions that interfere with the red cell turnover, such as hemolytic or iron deficiency anemia, chronic malaria, major blood loss, or blood transfusions, may result in spurious HbA1c values [17]. 

Recently, racial and ethnic disparities have been reported in the relationship between glucose levels and HbA1c [18]. Although the reasons for those differences are still unknown, several cut-off levels of HbA1c have been suggested to screen for diabetes in different ethnic populations. Since the etiology and significance of such disparities remain unclear, race-specific diagnostic values are currently not recommended [17]. Few studies have been conducted in Brazil to evaluate the efficiency of HbA1c in diagnosing diabetes and pre-diabetes. It is still largely unknown whether the performance of the recommended HbA1c cut-off value of ≥6.5% (48 mmol/mol) is suitable for the Brazilian population. Therefore, we conducted a population-based survey to examine the suitable cut-off values for HbA1c in the Brazilian population. Further, the homeostasis model assessment of insulin resistance (HOMA-IR) was also examined against HbA1c values to identify the most suitable cut-off points for HOMA-IR to predict the risk of diabetes.

## 2. Materials and Methods 

### 2.1. Study Population

A cross-sectional study was conducted between August 2012 and January 2013 in the city of Pindoretama, in the state of Ceará (CE), Northeastern Brazil. Subjects of both genders and age ≥20 years, who were able to communicate and wanted to join the study were considered eligible to be included. Pregnant women, those below 20 years of age, with an acute or chronic severe cardiac, renal, or hepatic illness, as well as physically or mentally disabled individuals unable to follow simple questions and examinations were excluded. 

The health registry list with the names of Pindoretama’s citizens in alphabetic order was applied to select eligible participants. Random numbers were generated with the statistical software R [19] and matched with the names in the list thereafter. Eight hundred and six randomly selected subjects were invited to participate. Out of these, seven hundred and fourteen agreed to join the study, corresponding to a participation rate of 88.6%.

### 2.2. Ethics

The study was conducted in accordance with the ethical principles outlined in the Helsinki Declaration [20]. Prior to any investigation, written or verbal consent was obtained from all subjects. In the case of illiteracy, verbal consent was assured by a local witness, who signed the informed consent, to secure the free participation of the subjects. The participants were informed of their rights to withdraw from the study at any stage or withhold their data from analysis. The privacy of the participants regarding the results of the tests and the collected data was assured. Those who were diagnosed with any clinical condition were referred to the respective health center for further follow-up. Before undertaking the research, the protocol was approved by both the local Ethical Committee in Brazil (Protocol Number: 045.06.12) and the Regional Committee for Medical and Health Research Ethics (REK) in Norway (Reference: 2012/779/REK sør-øst D).

### 2.3. Survey Procedures and Data Collection Tools

The selected subjects were invited to join the study by the local Community Health Workers (CHWs). By the time of invitation, the CHWs briefly informed the potential participants about the study purposes and methods of investigation. The data collection took place in the six main community health centers located throughout the city. Approximately one month was spent on the fieldwork in each center. Thus, the subjects could choose the most suitable day of the week to attend the survey. The participants were instructed twice (by the CHWs in person and by a phone call on the previous day of the data collection) to start fasting from 8 pm of the night before the survey procedures. After the participants’ arrival at the study place, the details about the research objectives and investigations were explained again. Those willing to participate were given informed consent. Ten milliliters of peripheral venous blood was collected for measuring FPG and lipids levels. Then, the subjects took a 75 g oral glucose load (according to the World Health Organization guidelines) to be prepared to the OGTT. Two hours after the glucose load, another venous sample was drawn. During these 2 h, the participants were questioned by trained interviewers, with pre-tested questionnaires regarding socio-demographic, economic, clinical, and nutritional data. Anthropometric measurements, blood pressure, and body fat percentage (BF%) were registered. 

The weight was taken with the subject standing in bare feet and in light clothing, by using a portable digital scale, calibrated before use and recorded to the nearest 0.1 kg. The height was measured by using a wall-mounted stadiometer, recorded to the nearest 0.1 cm, with the participant looking straight and in the erect position. The body mass index (BMI) was calculated as the weight in kilograms divided by the square of height in meters (kg/m^2^). The BF% was measured by a portable bipolar body fat analyzer (Omron^®^, Model HBF-306, Omron Healthcare, Inc., Illinois, United States). The waist circumference was taken by using a non-stretchable tape, placed horizontally on the midpoint between the lower part of the 12th rib and the top of the iliac crest, under the mid-axillary line. To the hip girth measurement, a similar tape was positioned to the maximum circumference around the buttocks. Waist and hip circumference were recorded to the nearest 0.1 cm. The waist-to-hip ratio (WHR) was calculated as the waist divided by the hip measurement. Following the recommendations of the World Health Organization (WHO), for males, a WHR ≥ 0.90 cm was considered “high” (a substantially increased risk of metabolic complications), whereas, for females, a WHR ≥ 0.85 cm was classified as “high” [21]. Blood Pressure was measured twice, within a 15 min rest interval, by using an electronic sphygmomanometer (Omron^®^ BP785 IntelliSense^®^ Automatic Blood Pressure Monitor with ComFitTM Cuff, Omron Healthcare, Inc., Illinois, United States). The mean value of the two measurements was applied in the analyses.

The blood samples were transferred to a sterile container, stored immediately over ice and centrifuged within approximately 1 h of collection. Plasma was frozen and transported to the laboratory, where the samples were stored at −20 °C until the analyses were performed. Fasting and 2 h plasma glucose levels were analyzed by the glucose oxidase method. Fasting insulin levels were determined by chemiluminescence. Total cholesterol (TC) was estimated by the cholesterol oxidase − phenol + aminophenazone (CHOD-PAP) method. High-density lipoprotein cholesterol (HDL-C) was determined by a homogenous enzymatic colorimetric method. The glycerol-3-phosphate oxidase − phenol + aminophenazone (GPO-PAP) method assessed the levels of triglycerides (TG). Low-density lipoprotein cholesterol (LDL-C) was estimated by the Friedewald Formula [22]. Capillary HbA1c levels were measured by A1CNow^®^ Multi-Test A1C System (Bayer). Quality control of the laboratory was assessed internally and externally.

Diabetes mellitus, impaired fasting glycemia (IFG), and Impaired glucose tolerance (IGT) were defined according to the 1999 WHO criteria. Thus, diabetes cases were those with fasting (venous) plasma glucose value ≥7.0 mmol/L (≥126 mg/dL), or the plasma glucose value 2 h after a 75 g oral glucose load ≥11.1 mmol/L (≥200 mg/dL), or both. Additionally, IGT was defined as fasting plasma glucose <7.0 mmol/L (<126 mg/dL), and 2 h plasma glucose ≥7.8 mmol/L (≥40 mg/dL), but <11.1 mmol/L (<200 mg/dL). IFG cases were defined as fasting plasma glucose ≥6.1 mmol/L (≥110 mg/dL), but <7.0 mmol/L (<126 mg/dL), and 2 h plasma glucose <7.8 mmol/L (<140 mg/dL). Subjects with IFG and IGT were classified as pre-diabetes cases. Dyslipidemia was defined as TG ≥1.7 mmol/L and HDL-C <0.9 mmol/L for men, and <1.0 mmol/L for women [23]. Insulin resistance in the fasting state was estimated with HOMA-IR ([insulin (mU/l) × glucose (mmol/L)]/22.5) [24].

### 2.4. Statistical Analysis

Statistical analyses were performed by using SPSS 25th version [25], R for Windows [19], and Stata 15th edition [26]. Numerical data were presented as means and 95% CI (confidence interval), while categorical data as numbers or percentages, and 95% CI. The overall prevalence rates of diabetes and pre-diabetes were adjusted for age and gender. Differences between the groups of means and proportions adjusted for age and gender were tested by logistic regression. The receiver operating characteristic (ROC) curve analysis was used to assess the discriminatory ability of the HbA1c test for detecting diabetes mellitus and pre-diabetes, given the WHO criteria as the gold standard. Additionally, ROC curves were also applied to compare the performance of HbA1c, FPG, and 2 h post-glucose load measurements for diagnosing diabetes. The ROC curves were achieved by using Stata [26]. Optimal cut-off points were obtained based on the highest Youden index [27]. The agreement for classification of diabetes using different cut-off points of HbA1c and the WHO criteria was assessed by the kappa statistic [28]. Bivariate Pearson’s correlation coefficient and bootstrapped 95% CI with 1000 replications was applied to evaluate the strengths of pairwise associations between HbA1c, FPG, and 2 h post-glucose load. Diagnostic test properties including sensitivity, specificity, positive predictive value (PPV), and negative predictive value (NPV), with 95% CI were also calculated for different cut-off points of HbA1c, FPG, and 2 h post-glucose load [29]. A *p*-value < 0.05 was considered statistically significant and all *p*-values presented were two-tailed.

## 3. Results

The clinical characteristics of 714 subjects classified as normal, pre-diabetics, or diabetics according to the WHO criteria are described in Table 1. 

In the study population, the adjusted prevalence rate of diabetes mellitus was 14.7% (95% CI: 12.2–17.2), and pre-diabetes 14.2% (95% CI: 11.6–16.7). The crude prevalence of diabetes was 16.1% (95% CI: 13.0–19.7%) among females, and 12.0% (95% CI: 8.4–16.7%) among males. The crude prevalence of pre-diabetes among females was 15.7% (95% CI: 12.7–19.3%), while among males was 11.2% (95% CI: 7.8–15.8%). Individuals with diabetes were more dyslipidemic and presented significantly higher WHR, BMI, BF%, systolic, and diastolic blood pressure than normal subjects. 

**Figure 1:**
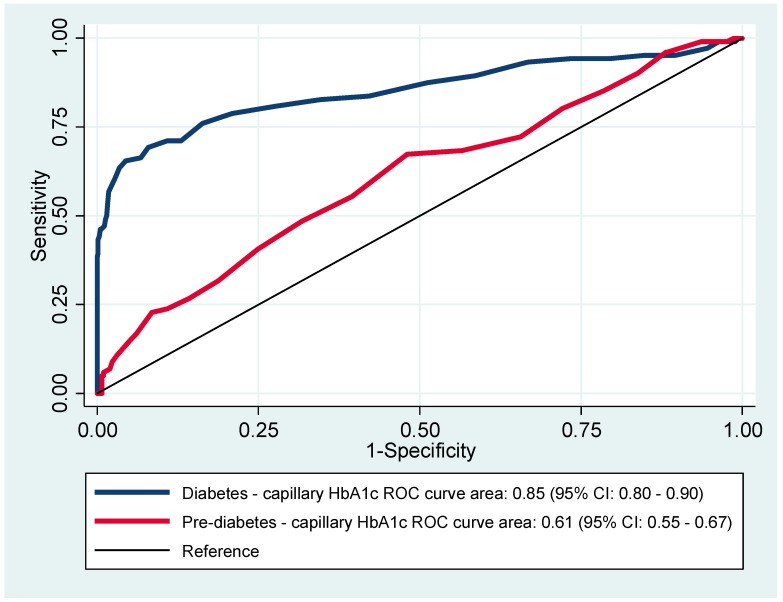
Receiver operating characteristics (ROC) curves for HbA1c (glycated hemoglobin) to diagnose diabetes and pre-diabetes.

The ROC curves for HbA1c to diagnose diabetes and pre-diabetes, considering the WHO criteria as the gold standard, are shown in Figure 1. 

The area under the curve (AUC) for detecting diabetes was 0.85 (95% CI: 0.80–0.90), and for pre-diabetes was 0.61 (95% CI: 0.55–0.67). According to the Youden index, the optimal HbA1c cut-off value for diagnosing diabetes was ≥6.8% (51 mmol/mol), and for pre-diabetes was ≥6.0% (42 mmol/mol). 

As described in Table 2, at the proposed point for diagnosing diabetes, i.e., capillary HbA1c ≥ 6.8% (51 mmol/mol), the sensitivity was 69.2%, specificity 92.1%, PPV 60.2%, and NPV 94.6%. At the cut-off for detecting pre-diabetes, i.e., capillary HbA1c ≥ 6.0% (42 mmol/mol), the sensitivity was 67.3%, specificity 52.0%, PPV 18.7%, and NPV 90.6%. 

**Figure 2:**
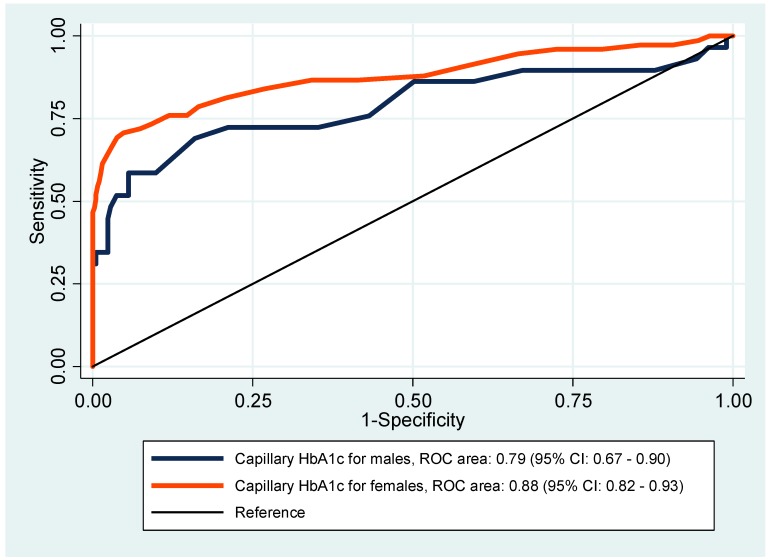
Receiver operating characteristics (ROC) curves for HbA1c to diagnose diabetes by gender.

Figure 2 presents the ROC curves for HbA1c to diagnose diabetes by gender.

The AUC for women was 0.88 (95% CI: 0.82–0.93), while for men was 0.79 (95% CI: 0.67–0.90). Nevertheless, there was no significant difference between the two curves (*p*-value = 0.18). Among women, based on the Youden Index, the most suitable HbA1c cut-off point for diagnosing diabetes was ≥7.0% (53 mmol/mol), while for men was ≥6.5% (48 mmol/mol). With HbA1c ≥ 7.0% (53 mmol/mol) for women, the sensitivity was 70.7% (95% CI: 59.0–80.6), and the specificity 95.2% (95% CI: 92.6–97.1). With HbA1c ≥ 6.5% (48 mmol/mol) for men, the sensitivity was 69.0% (95% CI: 49.2–84.7) and specificity 84.0% (95% CI: 78.4–88.7). A moderate agreement between the WHO criteria and the capillary HbA1c ≥ 6.5% (48 mmol/mol) for the classification of diabetes was found (Kappa = 0.46, *p* < 0.001). A higher agreement was observed between the WHO criteria and capillary HbA1c cut-off ≥6.8% (51 mmol/mol) (Kappa = 0.58, *p* < 0.001).

The diagnostic performances of FPG, 2 h post-glucose load, HbA1c ≥ 6.5% (48 mmol/mol) and ≥6.8% (51 mmol/mol) to diagnose diabetes are described in Table 3. 

The 2 h post-glucose load presented the highest sensitivity (77.1%, 95% CI: 67.9–84.8) among all others. At an HbA1c ≥ 6.5% (48 mmol/mol), the sensitivity (75.9%, 95% CI: 66.6–83.8) was higher than at an HbA1c ≥ 6.8% (51 mmol/mol) (69.2%, 95% CI: 59.4–77.9). However, the specificity (92.1%, 95% CI: 89.7–94.1), PPV (60.2%, 95% CI: 52.8–67.1) and accuracy (88.8 %, 95% CI: 86.2–91.0) were higher for the cut-off of HbA1c ≥ 6.8% (51 mmol/mol) than for the point of HbA1c ≥ 6.5% (48 mmol/mol).

**Figure 3:**
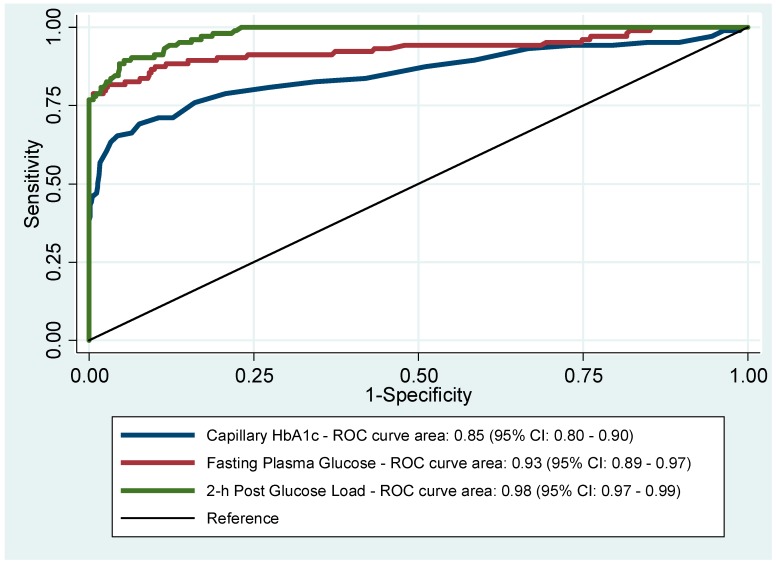
Receiver operating characteristics curves (ROC) for HbA1c, fasting plasma glucose (FPG), and 2 h post-glucose load to diagnose diabetes.

Figure 3 shows the ROC curves for HbA1c, FPG, and 2 h post-glucose load to detect diabetes, given the 1999 WHO criteria as the gold standard. 

The AUCs were 0.85 (95% CI: 0.80–0.90) for HbA1c, 0.93 (95% CI: 0.89–0.97) for FPG, and 0.98 (95% CI: 0.97–0.99) for 2 h post-glucose load alone. The Pearson’s correlation coefficient between HbA1c and FPG was 0.78 (95% CI: 0.70–0.83) and between HbA1c and 2 h post-glucose load was 0.86 (95% CI: 0.81–0.89).

**Figure 4:**
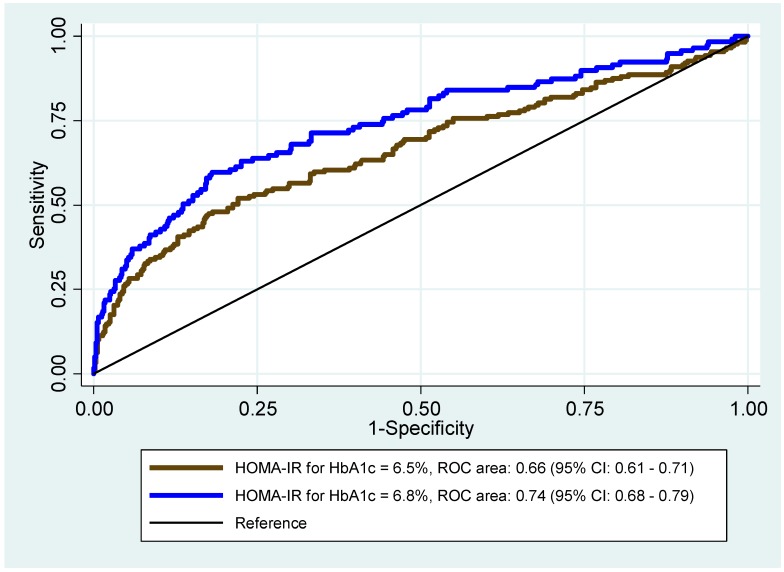
Receiver operating characteristics (ROC) curves for the homeostasis model assessment of insulin resistance (HOMA-IR) for cut-off values of HbA1c at 6.5% and 6.8%.

Figure 4 presents the ROC curves to identify the best cut-off value for HOMA-IR against the recommended cut-off point of HbA1c ≥ 6.5% (48 mmol/mol), and the proposed cut-off value of ≥6.8% (51 mmol/mol) from our data. 

The AUC for HbA1c at 6.5% (48 mmol/mol) was 0.66 (95% CI: 0.61–0.71), and for HbA1c at 6.8% (51 mmol/mol) was 0.74 (95% CI: 0.68–0.79). According to the Youden Index, the optimal HOMA-IR cut-off value for HbA1c at 6.5% (48 mmol/mol) was 1.81, and at 6.8% (51 mmol/mol) was 2.06. At the HOMA-IR cut-off point of 1.81, the sensitivity was 52.0% (95% CI: 44.4–59.5) and specificity 78.0% (95% CI: 74.2–81.5), while at the point of 2.06, the sensitivity was 59.7% (95% CI: 50.3–68.6) and specificity 81.9% (95% CI: 78.5–84.9).

## 4. Discussion

We found a higher prevalence rate of diabetes and pre-diabetes than previous large studies conducted in Brazil [30,31,32]. A large population-based survey, known as the Brazilian Multicenter Study, conducted on a representative sample (*n* = 21,847) of the urban population aged 30 to 69 years in nine large cities between 1986 and 1988, showed that the prevalence of DM was 7.6 and impaired glucose tolerance (IGT) was 7.8% [31]. More recently, between 1996 and 1997, another cross-sectional study conducted in Southeastern Brazil found that the overall prevalence of DM was 12.1% and IGT 7.7% [32]. 

Our study suggested that the optimal HbA1c cut-off value for diagnosing diabetes was 6.8% (≥51 mmol/mol), which is higher than the cut-off value of ≥6.5% (≥48 mmol/mol) recommended by the International Expert Committee [17] and the American Diabetes Association [12]. Even though the optimal HbA1c cut-off value for pre-diabetes found in this study, i.e., ≥6.0% (≥42 mmol/mol), was higher than that recommended by the ADA, i.e., ≥5.7% (≥39 mmol/mol) [12], it was similar to the cut-off value suggested by the International Expert Committee for high-risk groups, i.e., ≥6.0% (≥42 mmol/mol) [17]. Although our cut-off value of ≥6.8% (≥51 mmol/mol) showed a somewhat lower sensitivity compared to the recommended cut-off of ≥6.5% (≥48 mmol/mol), the PPV was substantially higher (60.2% versus 44.5%). 

Our proposed HbA1c cut-off value to diagnose diabetes was higher than the results found in China [33], Bangladesh [34], South Africa [35], Bulgaria [36], Norway [37], USA [38], and in a South Indian population [39]. To the best of our knowledge, no other previous studies suggested a higher HbA1c cut-off value to identify diabetes and pre-diabetes compared to the suggested guideline by the Expert Committee [17] and the ADA [12]. One of the very few studies conducted in Brazil about the topic has suggested a cut-off of HbA1c ≥ 6.0% (≥42 mmol/mol), which showed a sensitivity of 51.3% to diagnose diabetes [40]. 

Unlike many other populations, Brazilians compose one of the most heterogeneous societies in the world, as a result of five centuries of miscegenation between European colonizers, slaves from Africa, and autochthonous Amerindians. Since Brazil is a continental country with huge socioeconomic, ethnic and regional disparities, the findings in the present study may not be representative for the whole country. Caution should be taken when generalizing the results. Recently, it has been reported that racial and ethnic variations in HbA1c may impact its use as a diagnostic tool for diabetes. Studies have shown that adjustments for sociodemographic characteristics, access to healthcare, quality of care, and self-management behaviors attenuate, but does not fully explain the racial and ethnic differences in HbA1c. Therefore, some have argued that relying solely or preferably on HbA1c for the diagnosis of diabetes may lead to misclassification and systematic error. Factors such as differences in red blood cell survival, extracellular-intracellular glucose balance, and nonglycemic genetic determinants of hemoglobin glycation are now being investigated as potential contributors for ethnic disparities [18]. 

Although the International Expert Committee has recommended the use of HbA1c as a diagnostic tool for diabetes, its utility for identifying pre-diabetes has been considered problematic [17]. In our study, a large proportion of the subjects diagnosed with pre-diabetes using the WHO criteria had normal HbA1c levels. Moreover, we found a considerably lower AUC for pre-diabetes. Our results, therefore, do not support HbA1c as an adequate diagnostic tool for pre-diabetes in this population. 

In the AUC analysis, we found that 2 h post plasma glucose had the highest AUC in the diagnosis of diabetes, followed by FPG and HbA1c. Additionally, in agreement with previous studies, significant correlation coefficients between HbA1c and FPG, and between HbA1c and 2 h plasma glucose were demonstrated [33,39].

The homeostatic model assessment (HOMA), first described by Matthews et al. in 1985 [24], has been widely used particularly in epidemiological and clinical studies and proved to be a robust tool for assessing insulin resistance (IR) [41]. Nevertheless, the cut-off values of HOMA-IR greatly vary among different races, ages, genders, diseases, complications, and other factors [42]. Therefore, we attempted to measure the cut-off level of HOMA-IR against the recommended cut-off of HbA1c of 6.5% (≥48 mmol/mol) for the diagnosis of diabetes, as well as in relation to the best cut-off value of HbA1c found in the study, which was 6.8% (51 mmol/mol). The AUC for the ROC at HbA1c ≥ 6.5% (≥48 mmol/mol) was 0.66 (95% CI: 0.61–0.71), while for HbA1c ≥ 6.8% (≥51 mmol/mol) was 0.74 (95% CI: 0.68–0.79). The sensitivity and specificity were also higher for the HOMA-IR at 2.06 for the best fit of HbA1c at 6.8% (51 mmol/mol), compared to HbA1c at 6,5% (48 mmol/mol). These findings may also indicate an improved assessment of HbA1c at 6.8% (51 mmol/mol) and a cut-off value of 2.06 for HOMA-IR for the risk of diabetes and CVD in this population.

Among others, the cross-sectional design and sample size are limitations of our study. In order to determine the ideal cut-off points for diagnostic tests of diabetes, the ability of each method to predict the chronic complications of diabetes (such as diabetic retinopathy) should be explored. However, given the cross-sectional nature, we only evaluated the sensitivity and specificity of the various tools. Future follow-up studies are needed to provide more valuable conclusions. The ADA has recommended that, in the absence of unequivocal hyperglycemia, an abnormal HbA1c, fasting plasma glucose, or oral glucose tolerance test result that meets the criteria for diabetes should be confirmed by repeat testing before making a diagnosis of diabetes. Specially, when two different tests are available and the results are discordant, the test with a result above the diagnostic threshold should be repeated, and the diagnosis should be made on the basis of the confirmed test [12]. In our study, we were unable to retest and confirm blood glucose abnormalities. Further, HbA1c was measured from the whole capillary blood. However, a large body of scientific evidence suggests a high degree of sensitivity, specificity, and PPV between capillary blood and venous blood for HbA1c measures [43]. Known confounding factors such as hemoglobinopathies, severe iron deficiency anemia, hemolytic anemia, and renal or hepatic dysfunction may also have influenced our findings. 

Of strength, to the best of our knowledge, this was the first population-based study performed in Brazil to analyze the performance of HbA1c in diagnosing type 2 diabetes. In addition, the survey was conducted by trained and highly motivated staff. The fasting state of the participants was secured at three times: (a) orientation at inclusion, (b) telephone call by the study nurse the night before the test, and (c) on-site investigation. All blood collections, transportation, and storage were performed by trained laboratory personnel, and final analysis was performed at a certified laboratory. Quality control of the laboratory was assessed internally and externally.

## 5. Conclusions

In conclusion, an HbA1c threshold of ≥6.8% (51 mmol/mol) can be considered a relatively sensitive marker for the diagnosis of diabetes in this population, which differs from the suggested value of ≥6.5% (48 mmol/mol) by the International Expert Committee and ADA [12,17]. However, our data suggest that HbA1c values may be a weak parameter to identify pre-diabetes cases. The debate surrounding the role of HbA1c as a diagnostic test addresses the relative merits and disadvantages of glucose versus HbA1c and brings into focus many biological considerations as well as factors such as cost and accessibility. Early detection of diabetes through HbA1c is likely to be cost-effective as timely initiation of treatment will prevent complications. More studies are required, particularly long-term prospective studies, including all possible factors of influence, such as ethnicity, biological mechanisms, food habits, and lifestyle, in order to confirm our findings in such a multi-ethnic and multi-cultural society like Brazil.

## Figures and Tables

**Table 1:** Characteristics of 714 subjects classified as normal, pre-diabetics, or diabetics, according to the WHO criteria.

Characteristics	Normal	Pre-Diabetes (IFG and/or IGT)	Diabetes Mellitus
% (95% CI)	71.15 (67.95–74.35)	14.15 (11.61–16.68)	14.71 (12.17–17.24)
Age (years)	42.54 (41.19–43.89)	49.58 (46.55–52.60) *	53.25 (50.29–56.22) *
Sex (female), % (*n*)	63.4 (322)	73.3 (74) *	72.4 (76)
Waist (cm)			
Male	87.77 (86.07–89.48)	94.42 (89.92–98.93) *	95.95 (91.63–100.27) *
Female	88.29 (86.93–89.66)	90.87 (88.04–93.70)	98.45 (95.58–101.32) *^,†^
Hip Circumference (cm)			
Male	94.74 (93.48–96.00)	98.17 (94.83–101.50)	98.13 (94.94–101.33) *
Female	99.18 (98.04–100.33)	100.91 (98.54–103.28)	103.50 (101.10–105.91) *
WHR, Mean (95% CI)			
Male	0.93 (0.91–0.95)	0.96 (0.92–1.00)	0.98 (0.94–1.02) *
Female	0.89 (0.88–0.90)	0.90 (0.88–0.92)	0.96 (0.94–0.98) *^,†^
WHR (high), % (95% CI)	63.48 (59.66–67.30)	79.04 (70.98–87.10) *	95.49 (90.61–100.37) *^,†^
BMI (kg/m^2^)	26.20 (25.76–26.64)	27.34 (26.36–28.32) *	29.67 (28.69–30.65) *^,†^
BF%, mean (95% CI)	32.03 (31.43–32.63)	33.64 (32.31–34.96) *	35.70 (34.35–37.06) *^,†^
Family History of DM, % (95% CI)	35.66 (31.45–39.87)	36.37 (26.95–45.79)	63.90 (54.49–73.30) *^,†^
SBP (mmHg)	125.09 (123.45–126.73)	134.04 (130.38–137.69) *	133.80 (130.16–137.43) *
DBP (mmHg)	75.43 (73.94–76.93)	79.61 (76.27–82.94)	80.58 (77.27–83.90) *
FPG (mmol/L)	4.61 (4.48–4.76)	5.46 (5.10–5.81) *	9.73 (9.38–10.09) *^,†^
2 h Post-Glucose Load (mmol/L)	6.06 (5.82–6.30)	8.21 (7.66–8.76) *	16.23 (15.69–16.77) *^,†^
Capillary HbA1c, Mean (95% CI)	5.98 (5.90–6.07)	6.13 (5.94–6.31) *	8.11 (7.92–8.29) *^,†^
Fasting Insulin (micro UI/mL)	6.14 (5.71–6.57)	8.00 (7.03–8.96) *	8.63 (7.69–9.58) *
Total Cholesterol (mmol/L)	4.62 (4.54–4.71)	4.81 (4.62–4.99)	5.04 (4.86–5.23) *
Triglycerides (mmol/L)	1.32 (1.17–1.47)	1.88 (1.54–2.21) *	2.35 (2.01–2.68) *
HDL (mmol/L)	1.23 (1.22–1.24)	1.22 (1.20–1.24)	1.20 (1.18–1.23)
LDL (mmol/L)	2.84 (2.76–2.91)	2.91 (2.73–3.09)	2.90 (2.73–3.08)
Dyslipidemia, % (95% CI)	19.69 (16.17–23.21)	27.59 (18.80–36.37)	49.44 (39.60–59.27) *^,†^

Data are provided as mean (95% confidence interval) or percentage (95% confidence interval), adjusted for age and gender. Comparisons between the groups were performed adjusting for age and gender. * Significantly (*p* < 0.05) different from Normal. ^†^ Significantly (*p* < 0.05) different from Pre-Diabetes. WHO: World Health Organization. IFG: Impaired Fasting Glycemia. IGT: Impaired Glucose Tolerance. CI: Confidence Interval. WHR: Waist-to-Hip Ratio. BMI: Body Mass Index. BF%: Body Fat Percentage. DM: Diabetes Mellitus. SBP: Systolic Blood Pressure. DBP: Diastolic Blood Pressure. FPG: Fasting Plasma Glucose. HbA1c: Glycated Hemoglobin. HDL: High-Density Lipoprotein. LDL: Low-Density Lipoprotein.

**Table 2:** Diagnostic properties of different HbA1c cut-off levels for detecting diabetes and pre-diabetes, applying the WHO criteria as the gold standard.

Diabetes					Pre-Diabetes			
HbA1c %, (mmol/mol)	Sensitivity% (95% CI)	Specificity% (95% CI)	Positive Predictive Value % (95% CI)	Negative Predictive Value % (95% CI)	Sensitivity% (95% CI)	Specificity% (95% CI)	Positive Predictive Value % (95% CI)	Negative Predictive Value % (95% CI)
≥5.7 (39)	94.2 (87.9–97.9)	26.5 (23.1–30.2)	18.1 (17.1–19.1)	96.4 (92.4–98.3)	80.2 (71.1–87.5)	27.9 (24.0–32.0)	15.4 (14.0–16.9)	89.6 (85.0–92.9)
≥6.0 (42)	87.5 (79.6–93.2)	48.8 (44.7–52.8)	22.7 (20.9–24.7)	95.8 (93.1–97.4)	67.3 (57.3–76.3)	52.0 (47.5–56.4)	18.7 (16.3–21.3)	90.6 (87.9–92.8)
≥6.4 (46)	78.8 (69.7–86.2)	79.1 (75.6–82.2)	39.4 (35.1–43.8)	95.6 (93.7–96.9)	31.7 (22.8–41.7)	81.2 (77.5–84.5)	21.7 (16.5–28.0)	87.9 (86.3–89.3)
≥6.5 (48)	75.9 (66.6–83.8)	83.7 (80.5–86.5)	44.5 (39.4–49.8)	95.3 (93.5–96.6)	26.7 (18.4–36.5)	85.8 (82–4–88.7)	23.6 (17.3–31.2)	87.7 (86.3–89.0)
≥6.8 (51)	69.2 (59.4–77.9)	92.1 (89.7–94.1)	60.2 (52.8–67.1)	94.6 (92.9–95.9)	16.8 (10.1–25.6)	93.9 (91.4–95.8)	31.1 (20.6–43.9)	87.3 (86.3–88.3)

HbA1c: Glycated Hemoglobin. WHO: World Health Organization. CI: Confidence Interval.

**Table 3:** Diagnostic performances of FPG, 2 h post-glucose load and HbA1c to diagnose diabetes, using the WHO criteria as the gold standard.

Diagnostic Criteria	Sensitivity % (95% CI)	Specificity % (95% CI)	Positive Predictive Value % (95% CI)	Negative Predictive Value % (95% CI)	Accuracy % (95% CI)
FPG (≥7 mmol/L)	75.2 (65.9–83.1)	100 (99.4–100.0)	100	95.9 (94.4–97.0)	96.4 (94.7–97.6)
2 h Post-Glucose Load (≥11.1 mmol/L)	77.1 (67.9–84.8)	100 (99.4–100.0)	100	96.2 (94.7–97.3)	96.6 (95.0–97.8)
HbA1c (≥6.5%, ≥48 mmol/mol)	75.9 (66.6–83.8)	83.7 (80.5–86.5)	44.5 (39.4–49.8)	95.3 (93.5–96.6)	82.6 (79.6–85.3)
HbA1c (≥6.8%, ≥51 mmol/mol)	69.2 (59.4–77.9)	92.1 (89.7–94.1)	60.2 (52.8–67.1)	94.6 (92.9–95.9)	88.8 (86.2–91.0)

FPG: Fasting Plasma Glucose. HbA1c: Glycated Hemoglobin. WHO: World Health Organization. CI: Confidence Interval.
